# Diabetes Mortality in the Post-Pandemic Era: What Recent Global Burden of Disease Data Reveals About COVID-19’s Lasting Impact

**DOI:** 10.3390/epidemiologia7030077

**Published:** 2026-06-02

**Authors:** Kaustubh Wagh, Gerardo Chowell

**Affiliations:** 1 Department of Population Health Sciences, School of Public Health, Georgia State University, Atlanta, GA 30303, USA; 2 Department of Applied Mathematics, Kyung Hee University, Yongin 17104, Republic of Korea

**Keywords:** diabetes mortality, COVID-19, Global Burden of Disease, forecasting, public health, SDG 3.4, pandemic impact, IDDM, NIDDM

## Abstract

The COVID-19 pandemic disrupted diabetes care globally and created a complex bidirectional health crisis. Recent forecasting efforts using pre-pandemic data projected substantial increases in diabetes mortality through 2030, raising concerns about achieving Sustainable Development Goal (SDG) 3.4. However, these projections did not account for pandemic-related disruptions to health systems and chronic disease management. The newly released Global Burden of Disease (GBD) 2023 data, covering the pandemic period through 2023, now provide a comprehensive empirical reference for assessing COVID-19’s observed impact on diabetes trends. This perspective adopts a forecast reconciliation and interpretation approach by examining counterfactual pre-pandemic diabetes mortality projections alongside GBD 2023 data, thereby shedding light on how pandemic-era mortality diverged from pre-pandemic trajectories. Critically, we note that insulin-dependent diabetes mellitus (IDDM, Type 1) and non-insulin-dependent diabetes mellitus (NIDDM, Type 2) have distinct etiologies and pandemic vulnerabilities, a distinction this article addresses. The evidence is striking: by 2023, global diabetes deaths had already exceeded 2.0 million per year, surpassing the 2030 upper forecast bound of 1.91 million, seven years ahead of the forecast horizon. NIDDM was the primary driver, with deaths crossing 1.9 million per year in 2023. These findings underscore the urgent need to strengthen diabetes prevention and management strategies as the world recovers from the pandemic-era disruptions in health systems and chronic disease care.

## 1. Introduction

Diabetes mellitus represents one of the most pressing global health challenges of the 21st century. As a chronic metabolic disorder affecting more than 500 million adults worldwide [1,2], diabetes contributes significantly to premature mortality, disability, and healthcare costs. The trajectory of the diabetes epidemic has been closely monitored through forecasting studies designed to inform policy decisions and resource allocation. However, the COVID-19 pandemic introduced an unprecedented disruption to both care delivery and disease progression, raising critical questions about the accuracy of pre-pandemic projections and the long-term health consequences of the pandemic era. Recent systematic analyses have documented consistent and accelerating increases in the global diabetes burden. Ong et al. [3] found that global diabetes cases exceeded half a billion by 2021, with projections reaching 1.31 billion by 2050, while Lin et al. [4] estimated that diabetes deaths would reach 1.59 million by 2025, with the steepest increases in low- and middle-income countries. He et al. [5] projected that global type 2 diabetes prevalence would increase by 52% between 2021 and 2044, with the greatest increases in regions with low-to-middle socio-demographic index. These estimates make clear that the burden is predominantly driven by type 2 diabetes (NIDDM) and its modifiable risk factors.

Earlier, we published forecasts of global diabetes mortality through 2030, using historical data from the Institute for Health Metrics and Evaluation’s Global Burden of Disease 2019 study [6]. This analysis, based on 30 years of data from 1990 to 2019, employed multiple forecasting models to project diabetes-related deaths across age groups, geographic regions, and income classifications. The findings painted a concerning picture: diabetes mortality was projected to reach 1.63 million deaths annually by 2030, representing a 10% increase compared to the 2019 levels. These trends threatened progress toward Sustainable Development Goal 3.4, which aims to reduce premature mortality from noncommunicable diseases, including diabetes, by one-third by 2030 [7]. However, these forecasts had a critical limitation: they could not account for the profound disruptions caused by the COVID-19 pandemic, which began just months after the data cutoff date [6]. Healthcare systems worldwide experienced severe strain, routine diabetes care was interrupted, and the virus itself interacted with diabetes in complex and often deadly ways. The question remained: how would the pandemic alter the projected trajectory of diabetes mortality?

The release of the Global Burden of Disease 2023 study in October 2025 marks a watershed moment in our understanding of this question [8,9]. For the first time, comprehensive global data now extend through the pandemic years and into 2023, providing empirical evidence of COVID-19’s actual impact on diabetes burden. This perspective article examines three key questions: What did pre-pandemic forecasting reveal about the expected trajectory of diabetes mortality? What do the GBD 2023 data show about the pandemic’s actual impact? What are the implications for public health policy and global health targets in the post-pandemic era?

This article is presented as a perspective, which, as per the MDPI guidelines, showcases current developments with an emphasis on the authors’ personal assessment, rather than providing a comprehensive systematic review. The novel perspective advanced here is a forecast reconciliation and interpretation approach: we compare counterfactual pre-pandemic projections [6] calibrated on 1990–2019 GBD data against the GBD 2023 empirical outcomes [8,9], yielding interpretive conclusions that neither dataset could provide alone. To our knowledge, this is the first study to undertake this comparison for diabetes mortality using GBD 2023 data. No formal database search with inclusion/exclusion criteria was conducted as this was not a systematic review; all data are publicly available, aggregated, and de-identified.

This article adopts a forecast reconciliation and interpretation perspective, explicitly comparing counterfactual pre-pandemic projections of diabetes mortality, generated under the assumption of trend continuity, with realized post-pandemic mortality trajectories now observable in GBD 2023. By placing pre-COVID forecasts alongside empirical post-COVID outcomes, this study clarifies how pandemic-related structural disruptions altered previously projected trends and highlights the implications of these deviations for assessing progress toward Sustainable Development Goal 3.4 [7].

## 2. Pre-Pandemic Forecasting: Key Findings and Implications

Before presenting the forecasting results, it is important to distinguish between the two principal types of diabetes, as they have different etiologies and relationships with COVID-19. Type 1 diabetes (IDDM) results from autoimmune destruction of pancreatic beta cells leading to absolute insulin deficiency; it is not preventable, accounts for approximately 5% of all diabetes cases, and most commonly presents in childhood or early adulthood. Type 2 diabetes (NIDDM) arises from insulin resistance and relative secretory insufficiency driven by modifiable metabolic and behavioral factors, principally obesity, physical inactivity, and dietary patterns, and accounts for over 95% of cases globally. The pandemic affected these two conditions differently: NIDDM’s modifiable risk-factor base rendered it disproportionately sensitive to pandemic-era lifestyle disruptions and to SARS-CoV-2 infection-triggered new cases, while IDDM patients faced distinct harms, including acute metabolic decompensation (diabetic ketoacidosis), insulin supply disruptions, and reduced access to specialist care.

Our previous study [6] analyzed diabetes mortality data from 1990 to 2019 using six distinct forecasting approaches: ARIMA (autoregressive integrated moving average), GAM (generalized additive model), GLM (generalized linear model), Facebook’s Prophet model, and two ensemble n-sub-epidemic models (weighted and unweighted). These models were calibrated exclusively on pre-pandemic data and therefore generated counterfactual projections assuming trend continuity. This multi-model approach was designed to capture different aspects of diabetes mortality trends and to provide robust uncertainty estimates. Full methodological details, including AIC-based model selection, calibration period, and forecast horizon, are reported in [6]; data were sourced from Our World in Data (GBD 2019, 1990–2019).

The global findings were sobering. Across all models, diabetes deaths were projected to reach approximately 1.63 million annually by 2030 (95% prediction interval: 1.48–1.91 million), marking a 10% increase from 2019 [6]. As shown in Table 1, the GBD 2023 data revealed that these forecasts substantially underestimated the actual pace of increase: by 2023, global diabetes deaths had already exceeded 2.0 million per year, a 29.1% increase over 2019, surpassing the 2030 upper prediction interval seven years ahead of the forecast horizon. Type 2 diabetes (NIDDM) was the primary driver, having crossed 1.9 million deaths per year in 2023 (+32.3% vs. 2019), while Type 1 diabetes (IDDM) showed a much more modest increase of 3.9% over the same period, consistent with its non-modifiable autoimmune etiology.

Perhaps most concerning were the age-specific trends. While type 2 diabetes (NIDDM) has historically affected older adults most severely, the forecasts revealed alarming increases among younger populations [6]. Deaths in the 15–49 age group were projected to rise by more than 15% (forecast range: 128,000–140,400 deaths/year by 2030), while the 50–69 age group faced increases exceeding 30% (forecast range: 647,700–777,900 deaths/year). GBD 2023 confirms that both age groups are already tracking beyond these forecasts: by 2023, deaths in the 15–49 group had reached approximately 148,000 per year (+22.7% vs. 2019), already exceeding the entire 2030 forecast range; the 50–69 group reached approximately 753,000 per year (+29.5% vs. 2019). This shift toward premature mortality, driven predominantly by rising NIDDM among younger adults in rapidly urbanizing low- and middle-income countries, directly contradicts SDG 3.4’s goal of reducing deaths before age 70 from noncommunicable diseases [7].

Geographic disparities were stark. The Southeast Asia region was projected to experience the highest absolute burden, with approximately 530,200–654,400 deaths by 2030 (+>35% vs. 2019 [6]). GBD 2023 data show that the region has already far exceeded this: deaths crossed 705,000 per year in 2023 (+48.2% vs. 2019), surpassing the 2030 upper forecast bound by 2023. In contrast, the Eastern Mediterranean region, which had the highest forecast relative increase (+>40% by 2030), showed only a 9.7% increase by 2023, markedly below the forecast trajectory. This divergence likely reflects limitations in vital registration and data quality issues in the region, warranting cautious interpretation.

Income-based analyses revealed troubling inequities [6]. Low- and middle-income countries were projected to experience increases of 35% or more, while upper-middle-income countries faced even steeper rises, exceeding 50% in some models. GBD 2023 data confirm this trajectory: low- and middle-income countries reached approximately 936,000 deaths per year in 2023 (+35.6% vs. 2019), already near the upper forecast bound of 941,600. Upper-middle-income countries reached approximately 650,000 deaths per year (+27.7% vs. 2019), and high-income countries reached approximately 324,000 deaths per year (+28.3% vs. 2019), growing faster than the pre-pandemic central forecast of +11–12% suggested. This pattern is consistent with broader evidence showing that the sharpest increases in type 2 diabetes (NIDDM) mortality are concentrated in low and low-middle-socio-demographic-index regions [5].

These forecasts demonstrated that SDG 3.4 targets were increasingly unattainable without dramatic interventions [7], revealed that the burden of type 2 diabetes (NIDDM) was shifting toward younger, economically productive populations, and underscored the critical importance of prevention. The World Health Organization responded to these trends by establishing its first-ever global target for diabetes in 2022, signaling an institutional acknowledgment of the escalating crisis [4]. However, as noted in the study’s Limitations section, these projections assumed relative continuity in healthcare systems and disease trends [6]. They could not anticipate the massive global disruption that would begin in early 2020, just weeks after the data cutoff date.

## 3. The Pandemic’s Impact: Evidence from Global Burden of Disease 2023

The Global Burden of Disease 2023 study, published in October 2025 [8,9], provides the first comprehensive assessment of health trends through the pandemic years and into early recovery. Led by the Institute for Health Metrics and Evaluation (IHME) and involving more than 19,000 collaborators from 167 countries, GBD 2023 now allows for a direct empirical evaluation of how pandemic-era outcomes diverged from the pre-COVID counterfactual forecasts (Table 1).

The broader toll of the pandemic provides essential context. The WHO estimated that approximately 14.9 million excess deaths occurred globally between January 2020 and December 2021 [14]. A concurrent analysis by the COVID-19 Excess Mortality Collaborators produced an even higher estimate of 18.2 million excess deaths over the same period [14]. GBD 2023 reported that COVID-19 ranked as the leading cause of death globally in 2021, before dropping to 20th place by 2023 [8]. However, the collateral damage to chronic disease management, including diabetes care, persisted long after COVID-19 itself receded from the leading causes of death.

The most striking finding from GBD 2023 is that diabetes has risen to become the fifth leading cause of global disease burden, measured in disability-adjusted life years [10]. In absolute mortality terms, global diabetes deaths exceeded 2.0 million per year in 2023, a 29.1% increase over 2019 and surpassing the pre-pandemic 2030 upper forecast bound of 1.91 million, seven years ahead of the forecast horizon. IHME Director Dr. Christopher Murray noted “marked increases” in diabetes burden, with the disease “shooting up the list progressively with each decade”. Critically, unlike the trajectory of acute COVID-19 deaths, which declined sharply as the pandemic waned, diabetes mortality showed no such recovery by 2023 [8].

The pandemic’s impact on diabetes manifested through multiple interconnected mechanisms, with important differences by diabetes type. For NIDDM (Type 2), COVID-19 and diabetes exhibited a dangerous bidirectional relationship [12]. Systematic review evidence suggests that diabetes, predominantly NIDDM, accounted for approximately 9.5% of severe COVID-19 cases and 16.8% of COVID-19 deaths, with disparities linked to country income, healthcare access, and baseline diabetes burden [12]. The elevated severity risk in NIDDM appears to reflect not hyperglycemia alone but the coexisting comorbidity burden (obesity, hypertension, chronic kidney disease, cardiovascular disease) that frequently accompanies type 2 diabetes. Conversely, SARS-CoV-2 infection is associated with an increased risk of new-onset diabetes, predominantly of the NIDDM phenotype, potentially through inflammatory mechanisms that promote insulin resistance [3,17]. A meta-analysis reported a 64% higher risk of diabetes (RR = 1.64, 95% CI: 1.51–1.79) in patients with COVID-19 compared with non-COVID-19 controls [17]. For IDDM (Type 1), the pandemic introduced different vulnerabilities: disrupted insulin access, postponed specialist appointments, and the metabolic stress of SARS-CoV-2 infection contributed to elevated rates of diabetic ketoacidosis (DKA) and severe glycemic instability, particularly during pandemic peaks.

Disruptions in the healthcare system had cascading effects on diabetes management. During pandemic peaks, routine diabetes care was often postponed or canceled. Patients with existing diabetes experienced deteriorated glycemic control, with studies showing that higher HbA1c levels correlated with worse COVID-19 outcomes, creating a vicious cycle [12,18]. Delayed diagnoses meant that many cases of new-onset diabetes, primarily NIDDM, went undetected during critical periods, likely contributing to later complications and mortality. The shift to telemedicine, while beneficial in some contexts, created barriers for patients lacking technology access or digital literacy, disproportionately affecting the same low-income populations facing the highest NIDDM burden [5].

The pandemic is also associated with an acceleration of several NIDDM risk factors. Economic disruptions and lockdowns contributed to reduced physical activity, changed dietary patterns, and increased obesity rates [9]. Mental health impacts, including anxiety and depression, further complicate diabetes management. GBD 2023 documented significant increases in mental health burden, noting these were “partly attributable to COVID”, while acknowledging that the upward trend in depression and anxiety predated the pandemic [10].

GBD 2023 reveals that the pandemic’s impact was far from uniform. Middle-income countries experienced an “enormous increase” in noncommunicable diseases, including diabetes, cardiovascular disease, and stroke [15]. This pattern aligns with pre-pandemic forecasts showing that middle-income regions faced the steepest rises, but the pandemic appears to have accelerated these trends. Critically, middle-income countries accounted for approximately 81% of the estimated 14.9–18.2 million global pandemic excess deaths [13,14], precisely the regions where the pre-pandemic forecasts projected the steepest increases in NIDDM mortality [6].

GBD 2023 data now confirm what was previously a projection. Our pre-pandemic models forecast that annual global diabetes deaths would reach 1.48–1.91 million by 2030 [6]; by 2023, deaths had already exceeded 2.0 million, surpassing the upper bound of that interval by 7 years. The type-specific breakdown reinforces the IDDM/NIDDM distinction: NIDDM deaths crossed 1.9 million per year by 2023 (+32.3% vs. 2019), while IDDM increased by only 3.9% over the same period. The post-pandemic acceleration in NIDDM prevalence documented by Tang et al. [11] (annual % change rising from 2.90% pre-pandemic to 3.52% post-pandemic) provides one mechanistic explanation for why the realized trajectory exceeded the counterfactual forecast.

## 4. Public Health Implications and Policy Priorities

The convergence of pre-pandemic forecasts [6] and GBD 2023 pandemic-era data [8,9] creates a clear imperative for public health action. The divergence between counterfactual pre-COVID projections and realized post-pandemic trajectories indicates that diabetes mortality, driven overwhelmingly by NIDDM, is rising faster than anticipated, and that achieving SDG 3.4 targets by 2030 will require unprecedented interventions [7].

First, health systems must rapidly assess and address the accumulated deficits in diabetes care from the pandemic years. This includes identifying individuals whose diabetes diagnoses were delayed, patients whose glycemic control deteriorated during healthcare disruptions, and communities where preventive services were curtailed [12,15]. Catch-up screening campaigns, expanded access to diabetes medications, and intensive management programs for those with deteriorated control should be prioritized.

Second, prevention strategies must be strengthened, particularly in low- and middle-income countries experiencing the steepest increases [5,6]. The data clearly show that type 2 diabetes (NIDDM) is shifting toward younger, economically productive age groups in rapidly urbanizing regions. This demographic shift demands early interventions targeting workplace health, school-based nutrition programs, and community-level physical activity initiatives.

Third, pandemic preparedness planning must explicitly incorporate noncommunicable disease management [12,15]. Future pandemic response plans should include provisions for maintaining essential diabetes services, including insulin supply continuity for IDDM patients and screening programs for NIDDM, and for rapidly implementing telemedicine and remote monitoring capabilities.

Fourth, global health financing mechanisms must recognize the intersection of infectious and noncommunicable diseases. The pandemic demonstrated that these traditionally siloed domains are deeply interconnected: NIDDM increases vulnerability to infectious diseases, while infectious disease outbreaks disrupt diabetes care and may trigger new cases [9,12].

Fifth, the global community needs more timely and granular health data. GBD 2023, released in October 2025, provides data through 2023, a lag of nearly two years [8]. Investment in health information systems, particularly in LMICs, could enable more responsive policymaking. Additionally, forecasting methodologies should incorporate scenario-based approaches that account for potential disruptions, such as interrupted time-series or segmented regression models, rather than assuming linear continuity [6].

Finally, the diabetes crisis demands an honest acknowledgment of progress, or lack thereof, toward SDG 3.4 [7]. The evidence now clearly shows that the world is moving in the wrong direction on premature NIDDM mortality, particularly among working-age populations in the regions with the least resources. These findings warrant recognizing diabetesas a health security issue that requires emergency-level interventions in high-burden regions, consistent with the WHO’s recent establishment of global diabetes targets [19].

## 5. Limitations

Several limitations of this perspective and its data sources warrant acknowledgment. GBD 2023 estimates rely on vital registration data of variable completeness, particularly in LMICs, where systematic misclassification of diabetes as a contributing versus underlying cause of death is common, and modeling is used to fill data gaps, introducing uncertainty that is difficult to fully quantify. The cross-sectional and observational nature of the underlying evidence means that associations described throughout cannot establish definitive causation; language such as “is associated with”, “consistent with”, and “appears to” is used deliberately throughout. The pandemic years represent a structural break in mortality trends that simple trend extrapolation cannot fully capture; future forecasting efforts should consider interrupted time-series, segmented regression, joinpoint analysis, or scenario-based approaches rather than assuming trend continuity from either the pre- or post-2020 period. Finally, type-specific mortality data (IDDM vs. NIDDM) remain limited in LMICs, constraining type-differentiated analyses.

## 6. Conclusions

GBD 2023 data indicates that diabetes mortality has already moved beyond the upper range of pre-pandemic forecasts. Global diabetes deaths exceeded 2.0 million per year in 2023, surpassing the upper bound of the 2030 forecast [5,6]. NIDDM deaths exceeded 1.9 million per year in 2023 (+32.3% vs. 2019); the 15–49 age group exceeded its entire 2030 forecast range by 2023; and Southeast Asia surpassed its 2030 upper forecast bound, with deaths exceeding 705,000 per year. The pandemic is associated with an estimated 14.9 to 18.2 million excess deaths globally in 2020 and 2021 alone [13,14], and its after-effects continue to compound diabetes burden, particularly for NIDDM, whose modifiable etiology rendered it most sensitive to pandemic disruption. With limited time remaining until the SDG 3.4 deadline, and with 2023 mortality already exceeding the forecast ceilings in multiple categories, available data indicate that achieving a one-third reduction in premature noncommunicable disease mortality by 2030 is highly unlikely without extraordinary, coordinated intervention. Forecasting diabetes mortality serves not merely to predict the future, but also to motivate decisions that alter it [7,19].

## Figures and Tables

**Table 1:** **Pre-Pandemic Diabetes Mortality Forecasts vs. Pandemic Impact.** Column 3 shows the 2030 pre-pandemic forecast ranges across 6 models [6]. Column 4 shows Pandemic Impact through GBD 2023 estimated mortality in 2023 with percentage change versus 2019, and assesses whether the 2023 trajectory is consistent with, below, or already exceeding the 2030 forecast bounds. No new 2030 forecast based on GBD 2023 data has been generated; status assessments are directional interpretations only.

Category	Subcategory	Pre-Pandemic Forecast to 2030	Pandemic Impact (GBD 2023)
Global mortality	All diabetes	2019 baseline: ~1.55 M deaths/yr 2030 forecast range: 1.48–1.91 M deaths/yr; Central estimate: 1.63 M (+10% vs. 2019); 95% PI upper bound: 1.91 M deaths/yr	GBD 2023: >2.0 M deaths/yr|+29.1% vs. 2019. STATUS: Already exceeds the 2030 upper PI (1.91 M), 7 years ahead of the forecast horizon. Pandemic-era disruptions accelerated the trajectory beyond all pre-pandemic model estimates. Diabetes is now the 5th leading cause of global DALYs [10].
Diabetes type	Type 1/IDDM (autoimmune; ~5% of cases)	2019 baseline: ~78 K deaths/yr (est.); 2030 forecast range: ~80,100–92,900 deaths/yr (+>15% vs. 2019; upward trend all 6 models)	GBD 2023: ~81 K deaths/yr (est.)|+3.9% vs. 2019. STATUS: At the lower bound of the 2030 range; growing substantially more slowly than NIDDM, consistent with non-modifiable autoimmune etiology.
	Type 2/NIDDM (metabolic; >95% of cases)	2019 baseline: ~1.44 M deaths/yr (est.); 2030 forecast range: ~1.50–2.0 M deaths/yr (+>30% vs. 2019; primary driver all models)	GBD 2023: >1.9 M deaths/yr|+32.3% vs. 2019. STATUS: Already at the upper bound of the 2030 forecast range in 2023, 7 years early. Post-pandemic acceleration in NIDDM incidence (annual % change: 2.90% → 3.52% [11]) is the principal driver. Li et al. [12]: 16.8% of COVID-19 deaths had diabetes (predominantly NIDDM).
Age group	15–49 years (predominantly NIDDM)	2019 baseline: ~121 K deaths/yr (est.); 2030 forecast range: ~128,000–140,400 deaths/yr (+>15% vs. 2019) SDG 3.4 requires −33% vs. 2015	GBD 2023: ~148 K deaths/yr|+22.7% vs. 2019. STATUS: Already exceeds the entire 2030 forecast range (128–140 K) in 2023. The SDG 3.4 gap is confirmed by the data: the trajectory is +22.7% relative to 2019, whereas the target calls for a reduction. T2D burden in young adults doubled from 1990 to 2019 [4].
	50–69 years (predominantly NIDDM)	2019 baseline: ~582 K deaths/yr; 2030 forecast range: ~647,700–777,900 deaths/yr (+>30% vs. 2019), SDG 3.4 requires −33% vs. 2015	GBD 2023: ~753 K deaths/yr|+29.5% vs. 2019. STATUS: Within 2030 forecast range and tracking strongly toward upper bound (778 K); at current rate, upper bound likely exceeded before 2030. NIDDM comorbidities (obesity, CVD, CKD, hypertension) amplified COVID-19 severity in this group [12]. SDG 3.4 not on track.
WHO region	Southeast Asia (SEAR)	2019 baseline: ~476 K deaths/yr; 2030 forecast range: ~530,200–654,400 deaths/yr (+>35% vs. 2019; highest absolute burden any WHO region)	GBD 2023: >705 K deaths/yr|+48.2% vs. 2019. STATUS: Already exceeds the 2030 upper forecast bound (654 K) in 2023. Most severe divergence of any region. India, Pakistan, and Bangladesh primary drivers; limited healthcare infrastructure compounded pandemic-era care deficits.
	Eastern Mediterranean (EMR)	2019 baseline: ~122–128 K deaths/yr; 2030 forecast range: ~149,900–175,200 deaths/yr (+>40% vs. 2019; highest relative forecast increase any WHO region)	GBD 2023: +9.7% vs. 2019 (well below forecast trajectory) STATUS: Tracking markedly below forecast. By 2023, only +9.7% vs. 2019 against a forecast of +>40% by 2030. Possible explanations: incomplete vital registration and death underreporting, pandemic data quality limitations, or a genuine slower trajectory. This divergence requires further investigation and caution in interpretation.
Income class	Low-Middle Income (LMICs)	2019 baseline: ~690 K deaths/yr; 2030 forecast range: ~765,900–941,600 deaths/yr (+35–36% vs. 2019; highest absolute deaths any income group)	GBD 2023: ~936 K deaths/yr|+35.6% vs. 2019. STATUS: Near upper bound of 2030 forecast range (942 K) already in 2023; likely to exceed upper bound before 2030. 53% of 14.9–18.2 M global pandemic excess deaths concentrated here [13,14]; healthcare recovery slowest.
	Upper-Middle Income	2019 baseline: ~509 K deaths/yr; 2030 forecast range: ~533,700–768,200 deaths/yr (+>50% vs. 2019 in some models)	GBD 2023: ~650 K deaths/yr|+27.7% vs. 2019. STATUS: Within the 2030 forecast range, tracking toward central estimate. DALYs from major NIDDM risk factors (obesity, diet, inactivity) rose 30.7% since 2010 [9]; GBD 2023 confirms “enormous increase” in NCDs [15].
	High Income	2019 baseline: ~253 K deaths/yr; 2030 forecast range: ~245,700–336,900 deaths/yr (+11–12% vs. 2019; smallest relative increase)	GBD 2023: ~324 K deaths/yr|+28.3% vs. 2019. STATUS: Within and near upper end of 2030 forecast range; growing faster than pre-pandemic central forecast suggested, reflecting both pandemic excess and secular NIDDM trends. Better healthcare resilience versus LMICs.
SDG 3.4 target	Premature mortality (ages 30–70; approximated by 15–49 and 50–69 groups)	Required: −33% vs. 2015 by 2030 pre-pandemic forecast trajectories [6]: Ages 15–49: projected +23% vs. 2015 by 2030 Ages 50–69: projected +51% vs. 2015 by 2030 Both moving opposite to the SDG target	GBD 2023: Ages 15–49: +22.7% vs. 2019, already exceeding entire 2030 forecast range, SDG target completely off track, Ages 50–69: +29.5% vs. 2019, tracking to the upper 2030 forecast bound, SDG target completely off track. Conclusion: SDG 3.4 for diabetes is unlikely to be met without extraordinary intervention. GBD 2023 confirms this direction [8].

Abbreviations: IDDM: insulin-dependent diabetes mellitus (Type 1); NIDDM: non-insulin-dependent diabetes mellitus (Type 2); LMICs: low- and middle-income countries; DALYs: disability-adjusted life years; PI: prediction interval; CVD: cardiovascular disease; CKD: chronic kidney disease; SDG: Sustainable Development Goal; K: thousands; M: millions. For EMR, the 2023 figure warrants cautious interpretation, given the limitations of vital registration in that region. Sources: [3,4,6,8,9,10,11,12,13,14,15,16].

## Data Availability

No new primary data were generated. This perspective used publicly available, aggregated data from Our World in Data/IHME GBD 2019 and IHME GBD 2023, together with previously published forecast ranges from Wagh et al. [6].
